# Oral squamous cell carcinoma among Yemenis: Onset in young age and presentation at advanced stage

**DOI:** 10.4317/jced.50824

**Published:** 2012-10-01

**Authors:** Esam Halboub, Maha Al-Mohaya, Mahmoud Abdulhuq, Ahmad Al-Mandili, Yousef Al-Anazi

**Affiliations:** 1 Oral Medicine Department, Faculty of Dentistry, Damascus University, Damascus, Syria.; 2 Oral Medicine and Research Clinic, Medically Compromised Clinics and Research Unit, Department of Dentistry/Riyadh Military Hospital, Kingdom of Saudi Arabia.; 3 Oral Pathology Department, Faculty of Dentistry, Damascus University, Damascus, Syria.

## Abstract

Objectives: Oral cancer represents a health burden worldwide. Up to 90% of oral cancer cases are squamous cell carcinomas (SCC). The data on oral SCC in Yemen are lacking. The objective of this study therefore was to describe and analyze the demographic, clinical and histological characteristics of Yemeni patients with oral SCC. Study Design: In this cross-sectional study, two sets of retrospective data for Yemeni cancer patients were obtained officially by two different registries. Patients with oral SCC were included. Their ages were dichotomized using 40 and 45 years alternately as individual cut-points for young and old patients. The patients` demographic, clinical and histological characteristics were statistically analyzed. Results: There were 457 Yemenis with oral SCC; 253 patients (55.4%) were men. The overall mean age was 58.15±14.11 years. The tongue was the most affected oral sub-site accounting for 53% of the reported cases. The well and moderately differentiated oral SCC accounted for 55.5% and 25.6% of the total cases respectively. Noteworthy, 62 patients (14%) were affected by the age of ?40; this increased to 105 patients (23%) aged ?45 years. Additionally, a high proportion of oral SCC patients (62%, 283) were diagnosed at advanced tumor stages (regional extension or metastasized). The distributions of histological grades and tumor stages in young and old patients were significantly different (P=0.006 and 0.026 respectively). Conclusion: The relative frequency of oral SCC among Yemeni young people is high. Unfortunately, most of oral SCC patients in Yemen were diagnosed at advanced stage.

** Key words:**Oral squamous cell carcinoma, Yemen, young patients, advanced stage.

## Introduction

Oral cancer is an important global healthcare problem. Most of oral malignancies are squamous cell carcinoma (SCC) which has been reported to account for 70% to 90% (1). It develops primarily between the sixth and the seventh decades of life. Its` occurrence in young people (less than 45 or 40 years old) is rare (2,3); only 4% to 6% of all reported cases have been found in this sub-population (4,5). However, higher proportions of oral SCC (6,7), in addition to an alarming rise in its incidence, in young people are being documented worldwide (8,9).

Although oral cavity is accessible to visual evaluation, a high proportion of oral SCC is diagnosed at advanced stages and significantly more frequently in the developing countries (10). The more advanced the oral SCC the lower is the survival rate (6). Detection at early stage therefore will decrease the oral SCC-associated mortality and improve the patient`s quality of life.

A recent study revealed a quite high relative frequency of oral cancer among Yemenis, 3.7% (11), when compared to the populations of the neighboring countries. To the best of our knowledge, however, there are no studies in Yemen as yet on oral SCC per se. The current study, therefore, aimed to describe and analyze the demograp-hic, clinical and histological characteristics of Yemeni patients with oral SCC.

## Material and Methods

This cross-sectional study is based upon two sets of retrospective data combined together. They were provided officially by two different cancer registries. The first registry was the National Oncology Center (NOC), Sana`a, Yemen, which provided the data of all Yemenis who were registered with head and neck neoplasms in 2007 and 2008. The second registry was the Saudi Cancer Registry (SCR), Riyadh, Saudi Arabia, which provided the data of all Yemenis who were registered with neoplasms between 1994 and 2007 (11). The individual sets of data were provided anonymously and to be conditionally used for the research purposes only.

The data were coded, classified and tabulated as indicated elsewhere (11). According to the International Classification of Diseases for Oncology, third version (ICD-O-3) (World Health Organization, 2000), the primary oral sub-sites (topography) of the lip (C00), tongue (C01 and C02), gum (C03), flour of the mouth (C04), palate (C05) and other and unspecified parts of the mouth (C06) were included in the current study. For the included oral sub-sites, the cases with morphology (histology) of SCC, not otherwise specified (NOS) (807); Carcinoma, NOS (801); and Carcinoma, Undifferentiated, NOS (802)were grouped together as SCC.

Age of the included patients was dichotomized considering 40 and 45 years alternately as individual cut-points. Hence the patients aged 40 years or under were considered young patients and those aged over 40 years were considered old patients (12). Similarly, the second classification was based on 45 years (4). The descriptive statistics were presented as means (SD) and frequencies (%) as appropriate. The differences in age, by gender and country of registration, were analyzed by independent t-test while the associations of the clinical and histological factors with the gender and age category were analyzed using Chi-squared test. All analyses were performed at 0.05 significance value using IBM® Statistical Package for Social Studies (SPSS®) version 19.

## Results

There were 457 Yemeni patients with oral SCC; 281 patients (61.5%) were treated in Yemen and registered by the NOC. The others were treated in Saudi Arabia and registered by the SCR. There were 253 men (55.4%); the men to women ratio was 1.24:1. The overall mean age was 58.15±14.11 years (range: 16-110 years). The patients` age, by gender and country of registration, were comparable (data not shown).

The tongue was the most affected oral sub-site accounting for 53% of the reported cases. The well and moderately differentiated oral SCC accounted for 55.5% and 25.6% of the total cases respectively. Noteworthy, a high proportion of the affected patients were young; approximately 14% (62 patients) and 23% (105 patients) were affected by the age of 40 years or younger and 45 years or younger respectively. Additionally, albeit the tumor extension in 84 patients (18.4%) was unspecified, a high proportion of oral SCC patients (? 62%, 283 patients) were diagnosed at advanced stages (either regionally extended or metastasized).

The distribution of the tumor extension by gender was slightly different (P=0.047). The localized oral SCC was more frequent among men (59, 23.3%) compared to women (31, 15.2%). Similarly was the metastasized oral SCC (30 men [11.9%] versus 17 women [8.3%]). Contrarily, the regionally extended oral SCC was more frequent among women (112, 54.9%) compared to men (124, 49%) ( Table 1).

**Table 1:**
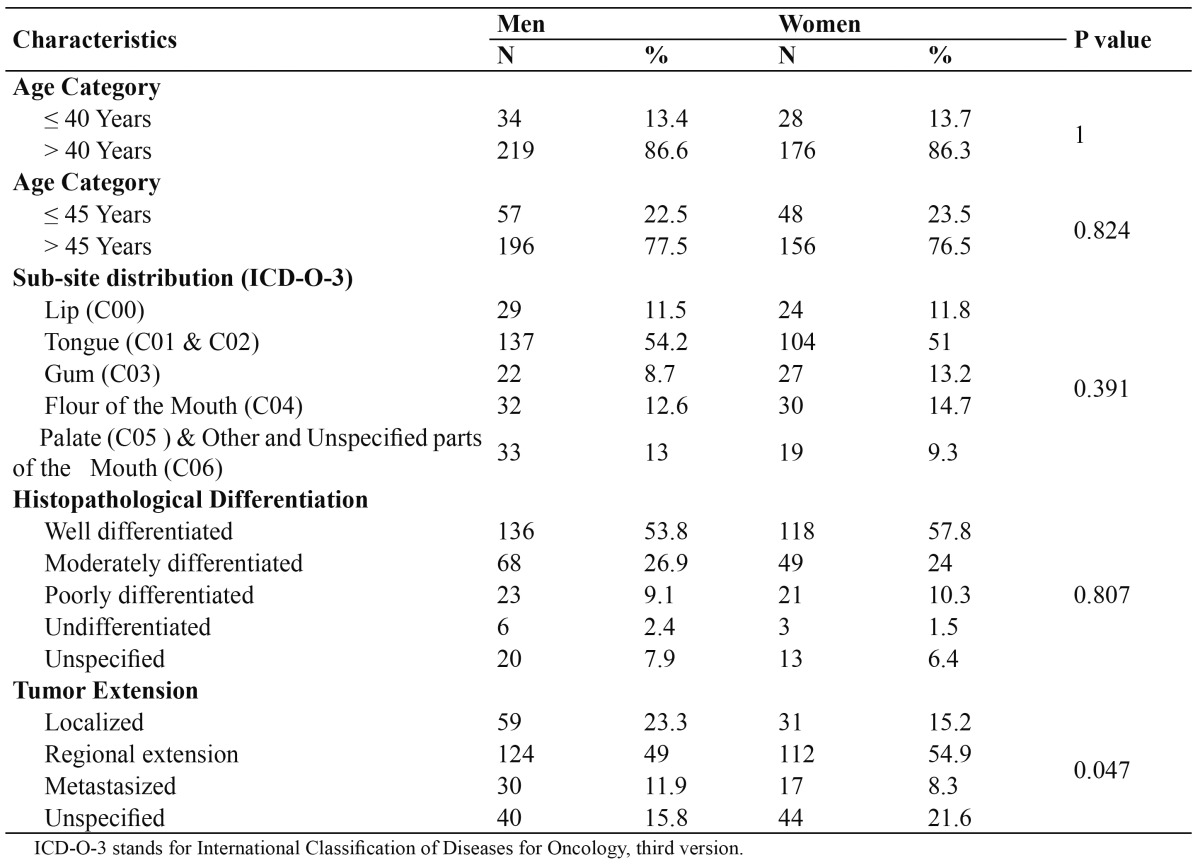
Distribution of 457 Yemeni patients with oral SCC by gender.

 Table 2 presents the clinical and histological characteristics of the young patients (? 40 years old) in contrast to the old patients (> 40 years old). The histologically undifferentiated oral SCC was more frequent among young patients (5, 8.1%) compared to old patients (4, 1%; P=0.006); the distribution of the other histological differentiation categories, however, were relatively comparable. Moreover, the oral SCC with regional extension or metastasis were more frequent among young patients (42, 67.7%) compared to old patients (241, 61%; P=0.026). Noteworthy, however, that the tumor extension in 9.7% and 19.7% of young and old patients respectively was unspecified.

**Table 2:**
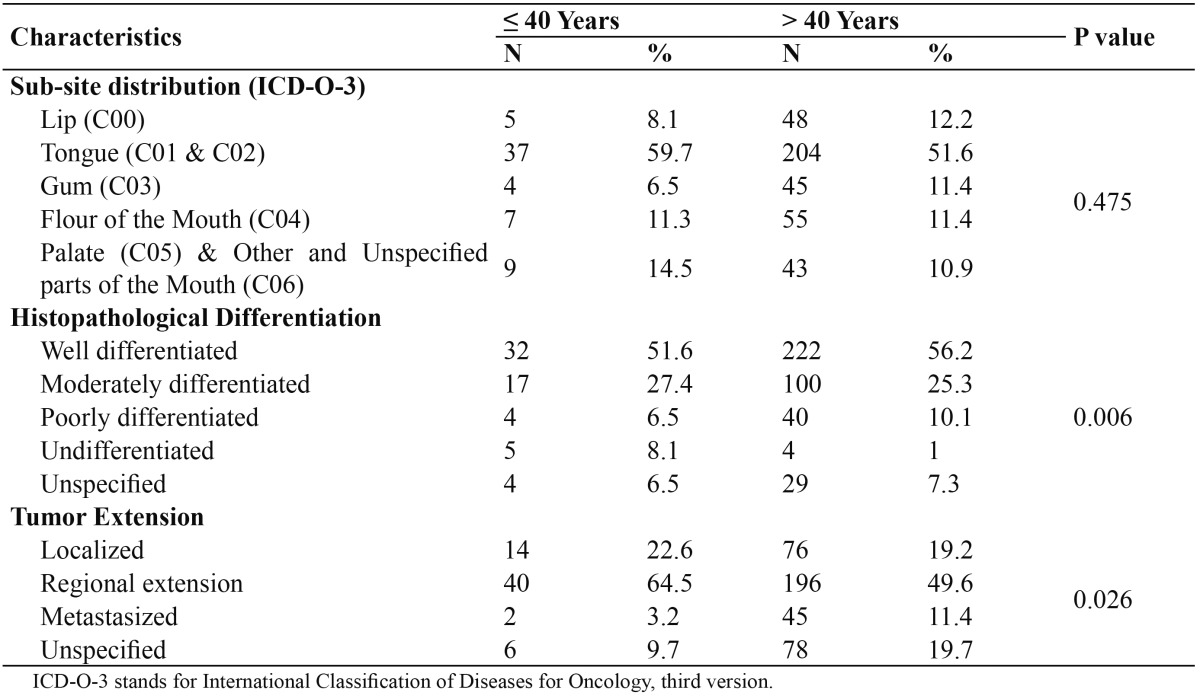
Distribution of 457 Yemeni patients with oral SCC by age categories; ?40 or > 40 years.

## Discussion

Development of oral SCC in young patients (younger than 40 or 45 years old) attracts attention worldwide. Ordinarily, oral SCC is a disease of aging with only 4-6% of the overall cases reported in the developed world affecting this sub-population (4,5). In the high incidence and other developing countries, however, higher proportions have been reported (6,13). The present study reported that 14% and 23% oral SCC affected Yemenis were young people (40 and 45 years old or younger respectively). Although comparable to what was reported in Iran (13% were younger than 40 years) (7), figure obtained from Jordan was much lower where only 16% of oral cancer were younger than 45 years (14). Moreover, even in some countries where oral cancer represents a real national burden such as Brazil (15) and Thailand (6), lower proportions of patients with oral SCC (12% and 13% respectively) were 45 years old and younger. Additionally, only 5.5% of the reported oral SCC patients in Sri Lanka were younger than 40 years old (5). On the other hand, slightly higher proportions were reported in India (16) and Myanmar (17); 17% and 30% of oral cancer patients were younger than 40 and 45 years old respectively. However, the highest proportion was reported in Nigeria; 30-40% of the oral SCC patients were younger than 40 years old (15,18).

It has been suggested that this may be a different sort of cancer as its biological behaviour is apparently more aggressive (19), though the reports have been scarce and conflicting (2,12,15). Moreover, a controversy does exist regarding exposure of young patients to the traditional risk factors for oral SCC, such as tobacco and alcohol (4,7,20). Although no data were available, practicing the different risk habits in Yemen (khat chewing, smoking and smokeless tobacco [locally known as shammah]) is not predicted to be different between young and old age groups. However, it is logic that young patients had been exposed for a shorter period. Hence they might have been practicing these habits more intensely; might have been exposed to other, still unknown, risk factors or both.

On the other hand, a recent increase in incidence of tonsilar and oropharyngeal squamous cell carcinoma among young patients was attributed to infection by Human Papilloma Virus (HPV) secondary to an increase in oral sex practice (21). However, the tonsilar and oropharyngeal sub-sites were not included in the present analysis and, more importantly, the oral sexual behavior is not predicted to be prevalent in reserved and religious communities like Yemeni people. Overall, the hypothesis of HPV implication should be considered seriously.

It is well known that presentation of oral SCC at advanced clinical stage predicts shorter survival and worse quality of life and poses much burden on the secondary and tertiary health care resources. In the present study, approximately 62% of Yemeni patients were diagnosed at advanced stages (either regionally extended or metastasized). This reflects low health knowledge and behaviour among Yemenis. Moreover, the specialized primary oral health care centers are scarce or even lacking. Last but not least, illiteracy, poverty and chronic debilitating diseases are prevalent in Yemen. Among populations with somewhat similar circumstances like Nigerians (22), Thais (6), Brazilians (15) and Iranians (23), high proportion of advanced oral cancer has been reported (100%, 73%, 65% and 50% respectively). On the other side, high proportion of oral cancer cases in the developed world are diagnosed at early stages (12,24).

More men were affected by oral SCC than women. This is a worldwide tendency (10). The cultural norms allow more men to be exposed to the proposed risk factors compared to women. In Yemen, khat chewing, smoking and shammah using could be considered men habits; prevalence and intensity of practicing these habits are much lower among Yemeni women. However, the men to women ratio in the present study is lower than that reported internationally, which highlights the burden of oral cancer to both genders equally.

The tongue was the most affected oral sub-site. A similar result has been reported in Yemen (25) and most of the developed and developing countries (10). There are geographic differences; however. In South Central Asia, the buccal mucosa, where betel quid is held, is the most affected oral sub-site (10,26). In Iraq (27) and Jordan (14) the lip is reported to be the most affected oral sub-site while the gingiva is the predominant oral sub-site in Nigeria (13). Although the tongue is not the main site correspondent to where khat, shammah or both are held, it is considered as one of the high risk areas for development of oral SCC.

In the current study, well and moderately differentiated oral SCC accounted for 81% of the reported cases. Comparable results were reported in Thailand (6) and Iran (7). Contrarily, high proportions of poorly differentiated oral SCC, 47%, were reported in Nigeria (13). Such differences could be attributed to the different preva-lence of the risk factors worldwide, intensity of exposure to these factors or both. The role of the genetic factors was suggested too (28).

It is difficult to explain the gender difference regarding the distribution of tumor extension. Indeed, this might not be a true difference as 15.8% and 21.6% of oral SCC in men and women respectively were not stage recorded.

Tumor staging and histological grading systems are good predictors of the prognosis and survival. Therefore, and in support of other studies (2,4), oral SCC in Yemeni young patients might be a different sort of cancer with worse prognosis as the histological differentiation and tumor staging are slightly worse compared to oral SCC in old patients. Contrarily, other reports showed that there are no specific clinical or pathological characteristics of oral SCC in young adults (6,12,15). At molecular level, nonetheless, even the comparable histological grades of oral SCC from these two age groups revealed different nuclear aberrations and proliferative activity. Therefore, Siriwardena et al. (28) emphasized the importance of conducting studies of genome-wide analysis, other than histopathological features, in deciding the prognosis and survival of these two important age categories. On the other side, as HPV-related oral and oropharyngeal carcinomas are mostly undifferentiated (21), again there might be an etiological role for this virus in development of undifferentiated oral SCC among young Yemeni patients.

In conclusion, the occurrence of oral SCC in young people, and its presentation at advanced stage in Yemen are relatively high. Therefore, intensive preventive and early detection and treatment programs are strongly recommended. Furthermore, large-scale case-control studies aiming to evaluate risk factors, placing great emphasis on the role of HPV, among young population in particular are suggested.
